# Who Should Decide the Outcome for a Clinical Trial? Comparing the Views of Stakeholders on Intervention Benefit Using Multi-Criteria Decision Modelling of Cognitive Remediation

**DOI:** 10.1093/schbul/sbag006

**Published:** 2026-04-21

**Authors:** Xiaoyu Zhang, Til Wykes, Huajie Jin, Dominic Stringer, Andrew Pickles, Matteo Cella, Alex Kenny, Emese Csipke, Imogen Kilcoyne, Jesus Perez, Uzma Zahid, Jonathan Wilson, Max Birchwood, Rose Tinch-Taylor, Rosa Ritunnano, Eileen Joyce

**Affiliations:** King’s College London, Institute of Psychiatry, Psychology and Neuroscience, Health Service and Population Research, London, SE5 8AF, UK; King’s College London, Institute of Psychiatry, Psychology and Neuroscience, Psychology, London, SE5 8AF UK; South London and Maudsley NHS Foundation Trust, London SE5 8AZ, UK; King’s College London, Institute of Psychiatry Psychology and Neuroscience, Health Services and Population Research, London, SE5 8AF, UK; King’s College London, Institute of Psychiatry Psychology and Neuroscience, Department of Biostatistics and Health Informatics, London SE5 8AF, UK; King’s College London, Institute of Psychiatry Psychology and Neuroscience, Department of Biostatistics and Health Informatics, London, SE5 8AF, UK; King’s College London Institute of Psychiatry Psychology and Neuroscience, Psychology, London, SE5 8AF, UK; South London and Maudsley NHS Foundation Trust, London, SE5 8AZ, UK; The McPin Foundation, London, E2 9DA, UK; King’s College London Institute of Psychiatry Psychology and Neuroscience, Psychology, London, SE5 8AF, UK; Essex Partnership University NHS Foundation Trust, Wickford, SS11 7XX, UK; Cambridgeshire and Peterborough NHS Foundation Trust, Cambridge, CB21 5EF, UK; King’s College London, Institute of Psychiatry Psychology and Neuroscience, Psychology, London, SE5 8AF, UK; Norfolk and Suffolk NHS Foundation Trust, Research and Development, Norwich, NR1 2DH, UK; University of Warwick Faculty of Medicine, Medical School, Coventry, CV4 7AL, UK; King’s College London Institute of Psychiatry Psychology and Neuroscience, Biostatistics and Health Informatics, London, SE5 8AF UK; University of Birmingham College of Life and Environmental Sciences, Institute for Mental Health, Birmingham, B15 2TT UK; University College London, Institute of Neurology, London, WC1N 3BG, UK

**Keywords:** clinical trial outcomes, stakeholder values, patient values, multi-criteria decision modelling, composite outcomes, cognitive remediation therapy

## Abstract

**Background and Hypothesis:**

Trials rarely include outcomes co-developed with stakeholders, which creates uncertainty about whether trial endpoints reflect the benefits valued by patients and clinicians. There is therefore a need to explore how stakeholder perspectives might influence the interpretation of trial results.

**Study Design:**

In a 2-phase study, we first engaged service users and staff to rank outcomes from a recently completed randomized controlled trial of cognitive remediation therapy (CRT). We then used multi-criteria decision modelling to generate value-weighted composite scores and reanalyzed the trial data from three arms: individual CRT, group CRT, and treatment as usual (TAU). This approach allowed us to examine how weighting outcomes differently, according to stakeholder perspectives, might affect the conclusions drawn from the trial.

**Study Results:**

Both stakeholder groups prioritized the Global Assessment Scale (primary outcome) and quality of life, but disagreed on the importance of cognition. Reanalyses using weighted composite scores produced the same robust finding as the original trial: CRT delivered in group or one-to-one formats provides significant benefit compared with TAU. Sensitivity analyses applying different stakeholder weights showed the same pattern. However, exploratory analyses revealed that group treatment for the service user weighted composite was not significant.

**Conclusions:**

This study shows how stakeholder values can identify nuances in outcomes depending on the value placed on treatment benefits. Embedding this approach into trial design can strengthen the alignment of outcomes with patient and service priorities and help close the gap between randomized trials and service implementation.

## Introduction

The criteria being used to judge the success or failure of mental health treatments are coming under increasing scrutiny. They are usually evaluated in randomized controlled trials (RCTs) with the benefit measured by a single clinical outcome, called the primary outcome. The power of the study to detect differences between the intervention and the comparison group or groups then depends on this single outcome and determines the number of individuals who need to be recruited as participants. All other outcomes are known as secondary and are downgraded in terms of their importance in evaluating clinical benefit. This view has been challenged particularly as those who are participants and potential intervention beneficiaries sometimes claim that the primary outcome would not have been the one they would have chosen.^1–4^ Service users often prefer co-designed patient reported outcome measures, but they are rarely the trial primary outcome.^5^

Clinicians charged with providing the intervention may also have emphasized different outcomes than researchers or service users. Information about preferred outcomes is available^6^ as is evidence of the shortcomings of popular primary outcome scales.^7^ The obvious solutions are to choose measures with service-users or use co-developed outcomes, but these are rarely used as primary outcomes in randomized trials^5^ despite their utility.^8–10^

We therefore need to consider other options for determining the value of interventions to both service users and service providers. This information may aid the implementation of interventions by considering potentially differing views of the benefit and is one step toward a shared decision-making approach that actively involves both patients and healthcare providers in treatment plans.

Different models have been suggested for enhancing the choice of measure of benefit using ratings of outcome importance given mainly by patients prior to a trial or to allow the assessment of benefit following the trial.^11–13^ For instance, we might use a broad set of outcomes that can be assigned relative weights and formed into a weighted composite outcome.^14^ One method for producing this is multi-criteria decision modelling (MCDM). This method integrates objective data collected as part of an RCT with the subjective values of stakeholders and allows us to understand not only the importance of different outcomes but what benefits would have been realized if stakeholders’ values had been used.^15^ Our adopted MCDM definition is a method “for appraising alternatives on individual, often conflicting criteria, and combining them into one overall appraisal”.^16^

There are likely to be differences in the level of importance attributed to an outcome measure between service users, providers and researchers and differences within groups too. For example, researchers will want to see a “clinical difference” measured in standard ways so their research leads to publications and will be acceptable to research funders and regulators. Service managers may prefer service costs to indicate a lower care burden whereas direct care staff might concentrate on symptoms and functioning. In contrast, people with lived experience are often concerned with the side effects of treatment or their distress^12,17^ or agree with direct care staff on the need to consider functioning outcomes.^18^ A user’s judgment of the importance of potential treatment effects is also known to be influenced by their demographic characteristics. Age, race, and household income level affected the weight given to outcomes in cardiovascular studies. For instance, younger people gave more weight to death than myocardial infarction or stroke whereas for older people it was the reverse.^19^ Other studies in schizophrenia also describe how life context affects the choice of outcomes.^20,21^

Given these different perspectives we compared how different stakeholder groups—people with lived experience and clinicians—prioritize outcomes from a clinical trial of cognitive remediation for first episode psychosis.^19^ This trial was chosen because despite large numbers of studies suggesting a benefit and its inclusion in some treatment guidance, there is still some dispute about the strength of the evidence.^20–22^ The trial primary outcome (Goal Attainment Scale^23^) was chosen following consultation with service users and providers, but there may be other outcomes that contribute to an overall view of benefit and the likelihood of implementation. This study evaluates the level of importance given to these different outcomes but also uses MCDM methods to investigate whether the trial effects might have differed if one group or another or all could have provided a more nuanced approach to detecting benefit. The aim is twofold. First to test whether different stakeholders make different decisions about the relative importance of potential outcomes, and second to test whether by using these importance rankings in a composite score would produce a different outcome in terms of the benefits of cognitive remediation.

## Methods

### Overall Design

The study is divided into 2 phases. The first assesses the perceived importance of the different measures used as primary and secondary outcomes in the CIRCuiTS^TM^ Trial. This 6 site, RCT recruited 377 participants from Early Intervention Services (EIS) to one of four arms. These were treatment as usual (TAU) and 3 ways of implementing therapy (one-to-one, group, and independent) to differentiate the level of therapist support needed for a successful outcome. Group and one-to-one therapy both significantly improved the primary goal, Goal Attainment Scale, which measures personal goal achievements in addition to improving cognition. Those 2 implementation methods could not be distinguished from one another. Phase 1 investigated the importance rankings, specifically between and within group differences of 3 groups: service managers who can choose interventions for their service, direct care staff who might provide the intervention and service users who could be in receipt of the intervention. Importance rankings were then used in phase 2 to construct weighted composites to reanalyze the data from the substantive trial to detect whether the treatment benefits would have been different using an MCDM analysis. Ethical approval for the study was provided by West of Scotland National Health Service (NHS) Research Ethics Committee (REC) 5 21/WS/0153.

### Involvement of Those With Lived Experience

The new Consolidated Standards of Reporting Trials (CONSORT) 2025 ^24^ now suggests including how lived experience was involved in the trial. The CIRCuiTS^TM^ trial, where data for this study are gleaned, involved people with lived experience in the design, methods and as authors in the main trial paper.^19^ We continued to discuss the MCDM method with this advisory group including the design and method of data collection. The design issues influenced by these advisors are described in the text. We also asked the Maudsley BRC FAST-R group of service users and carers to provide feedback on the protocol and all patient facing materials.^25^ Some original trial advisors are also authors of this paper.

### Phase 1—Importance Ranking of Outcomes Measured in the CIRCuiTS^TM^ Trial

#### Design, Recruitment, and Participants

A simple method for importance ranking was adopted in order that all people with a diagnosis of schizophrenia, some of whom may have cognitive difficulty, could easily take part.^26^ This was advised by our Patient and Public Involvement (PPI) panels. Importance rankings were collected through an online survey circulated to EIS staff and their clients or introduced by researchers visiting those services. Participants were asked to put the eight outcomes measured from the RCT in the order of the most important (score 8) to least important (score 1) by moving the most important to the top of the list, followed by the next most important and so on. No participant had any difficulty with this process. Three groups were surveyed—(1) people with lived experience of first episode psychosis who fulfilled the same criteria as the participants in the trial, (2) team managers in first episode services (NHS grade 7 and above), and (3) staff who delivered direct patient care (NHS grade 6 and below). All participants were recruited from July 21 to November 23 after the CIRCuiTS^TM^ trial had concluded. No participant had been involved in the original trial.

#### Measures

Participant characteristics (age, gender, ethnicity) were captured and for staff we additionally asked how long they had been in post.

The eight ranking characteristics and how they were measured in the RCT are:

1. *Satisfaction with therapy.*^27^

2. *Achieving service users goals*—the Goal Attainment Scale.^23^

3. *Increasing the number of things an individual spends their time on after therapy*—measured using the Time Use Schedule.^28^

4. *Improvement in thinking skills*—a global score from cognitive tests.^29–32^

5. *Increasing self-esteem*—Rosenberg Self Esteem Schedule.^33^

6. *Reducing positive symptoms*—from the PANSS.^34^

7. *Improving negative symptoms*—from the CAINS.^35^

8. *Improving overall quality of life*—the EuroQol.^36^

Further information on each measure is provided in the report of the original trial.^19^

#### Data Analysis

As the ranking data were not normally distributed, we used non-parametric tests for analysis. We compared outcome importance rankings across the three groups (people with lived experience, direct care staff, and team managers) using the Kruskal–Wallis test. If no statistically significant difference is found between staff groups, then we will combine the groups and compare them with the group of people with lived experience using a Mann–Whitney U test. If significant differences between staff groups are observed, we will retain the three-group comparison framework.

We investigated whether different groups agreed on the importance given to the outcomes. We calculated cross-group variability in ranking quantified using inter-quartile ranges (IQRs) and median absolute deviations (MADs) and then conducted Brown–Forsythe tests to formally compare the variance across groups.^37^ We were also interested in how participant characteristics affected their views and so conducted within group analyses to ascertain whether age, gender, and ethnicity affected importance rankings. Each group was stratified by age (median split), gender (men vs women), and ethnicity (white vs other), and differences considered using Mann–Whitney U tests. We also compared age, gender, and ethnicity distributions across respondent groups using Mann–Whitney U tests (for age and gender) and chi-square tests (for ethnicity).

#### Results

Table 1 summarizes the demographic data for the 92 participants in the ranking study. The lived experience sample is representative of the sample of participants in the RCT in each parameter.^19^ For the staff groups, as expected, those who are team managers have been working in the services for longer than direct care staff but otherwise have similar characteristics. Managers were mostly lead social workers, nurse managers, doctors, and clinical psychologists, whereas direct care staff were assistant psychologists, nurses, and trainee clinicians. Those with lived experience differed from staff as they were younger and fewer were of white ethnicity (see Table S1, age: *z* = 4.26, *P* < .001; ethnicity χ^2^ = 17.22, *P* < .001).

**Table 1:** Demographic Characteristics of the Participant Groups

Characteristic	Overall (*n* = 92)	Direct care staff (*n* = 31)	Team managers (*n* = 31)	People with lived experience (*n* = 30)
	No., % or Mean (SD) or Mean (SD); min to max			
**Gender**—female	54 (58.7)	21 (67.7)	23 (74.2)	10 (33.3)
**Age in years and age range**	35.5 (10.7) 18-64	38.5 (12.7) 24-64	38.9 (8.8) 28-63	28.8 (6.7) 18-41
**Ethnicity**				
Asian, Asian British or Asian Welsh	12 (13.0)	2 (6.5)	8 (25.8)	2 (6.6)
Black, Black British, Caribbean or African	21 (22.8)	6 (19.4)	0 (0.0)	15 (50.0)
Mixed or Multiple ethnic groups	6 (6.5)	1 (3.2)	2 (6.5)	3 (9.9)
White	47 (51.1)	21 (67.7)	20 (64.5)	6 (20.0)
Other ethnic group	6 (6.5)	1 (3.2)	1 (3.2)	4 (13.3)
Length of time in months staff members have worked in EIS service	N/A	27.5 (31.1)	46.2 (53.6)	N/A

#### Importance Rankings

There were no significant differences between team managers and direct care staff in the overall importance of an outcome (see Tables S2 and S3) so we combined them into a single “staff” group for further analyses. The importance ranking is shown in Table 2. In the overall scores, 2 outcomes were rated as the most important: improving a patient’s goals and improving quality of life, with 21% and 25% rating these outcomes of highest importance. The least supported were increasing self-esteem (7%) and reducing negative symptoms (5%) (see Tables S4 and S5). There was variation both within and between groups on the views of importance (see Table S6 and statistical differences in Table S6). The dispersion of importance rankings also differed across groups. Service users showed the most consistent rankings (Mean IQR = 3.25; Mean MAD = 1.69), while team managers exhibited the greatest variability (Mean IQR = 3.88; Mean MAD = 1.75). Brown–Forsythe tests found no significant variance differences for most outcomes, except for “reducing positive symptoms”, where variance differed significantly. No staff versus lived experience comparison was significant.

**Table 2:** Mean and Standard Deviation of Importance Rank by the Different Groups 8 is Most Important, 1 Least Important

Outcome item	Overall	Staff	Service user	Mann–Whitney U test *P*-value
	Mean (SD)			
1. Satisfaction with therapy	3.5 (2.6)	3.2 (2.6)	4.2 (2.6)	.054
2. Achieving the service user’s individual goals	5.2 (2.3)	5.16 (2.3)	5.3 (2.4)	.787
3.Increasing the number of things an individual spends time on	4.1 (2.1)	3.7 (2.0)	5.0 (2.1)	**.006** ^**a**^
4. Improvement in thinking skills	4.8 (2.0)	4.7 (2.0)	5.0 (1.8)	.514
5. Increasing self-esteem	4.3 (1.9)	4.6 (1.8)	3.7 (2.1)	**.032** ^**a**^
6. Reducing positive symptoms	4.9 (2.3)	4.6 (2.2)	5.4 (2.2)	.115
7. Improving negative symptoms	4.4 (2.1)	4.7 (2.0)	3.9 (2.2)	.103
8. Improving overall quality of life	5.0 (2.6)	5.6 (2.5)	3.6 (2.2)	**<.001** ^**a**^

^a^Significant effect.

We found significant differences on three outcomes: service users placed significantly higher importance on “increasing the number of activities” (*P* = .006); staff placed significantly greater importance on “improving self-esteem” (*P* = .032); staff also rated improving overall quality of life more highly (*P* < .001). The remaining 5 outcomes showed no statistically significant group differences (Table 2).

We also investigated potential demographic effects on the importance rank. When looking across the total group there were significant effects of age and ethnicity on the importance of increasing activity, and ethnicity also had an effect on the importance of self-esteem (Tables S8-S10). Within-group analyses also revealed a significant difference in the importance of increasing activities in the combined staff group with older staff rating it as more important (Tables S11-S13). There was no effect of gender on any importance ranks.

#### Discussion

The primary outcome in the CIRCuiTS^TM^ RCT was achieving service user’s goals and was one of the highest rated outcomes for all groups so the choice of outcome, despite being one not often used in this context, seems an appropriate measure of benefit and validates the original service user consultation. Another highly ranked outcome was quality of life that is often a secondary outcome in trials. Staff and users did differ but for 5 of the 8 outcomes there was no difference. Service users wanted more activities, and staff were more interested in quality of life and self-esteem. Positive symptoms were reasonably highly rated by both staff and service users and often reported as outcomes in trials as they interfere with life in the community. When they are prominent, they may necessitate admission to hospital and so it is not surprising that both groups have this emphasis. However, the lower score given by service users to self-esteem was unexpected but may be a result of the format of the importance ranking. If you have goals and quality of life at the top of your list that means that self-esteem must take a lower position. Demographic differences had few effects on the importance ranking although increasing activities seemed to be more consistently affected by age and ethnicity. These were the variables that were different between staff and service users (Table S1). The groups were too small to be confident in post hoc analyses, but it does suggest that demographic differences in the participant sample might affect their view of the trial benefits. Further research might point to the specific demographic issues that might affect the value participants place on an outcome domain.

### Phase 2—Multiple Criterion Decision Modelling

#### Method

##### Data

The intervention data are from an adaptive RCT starting with 4 trial arms, 3 active treatment arms differing in the amount of therapist support (independent, group, and one-to-one) for the software CIRCuiTS^TM^ and TAU. Data were collected at 3 time points with post-treatment data (week 14) as the primary endpoint. Following an interim analysis, recruitment was discontinued to one treatment arm and TAU. The primary outcome was the Goal Attainment Scale (GAS) with secondary outcomes listed in phase 1. The trial demonstrated benefits to cognition and GAS at post-treatment. The statistical analysis plan determined the comparisons and their order for this study (see^19^ supplement statistical analysis plan).

##### Design and Data Preparation

Individual participant rankings of the outcomes were synthesized into a consensus group ranking using a frequency-based aggregation procedure. For each rank position, the outcome most frequently assigned to that position across participants was selected. In cases where 2 or more outcomes had the same highest frequency for a given rank, the tie was resolved by examining the full rank distributions of the tied outcomes and assigning the outcome with the higher median rank (ie, indicating higher perceived importance) to that position.^38^ “Satisfaction with therapy” was not included in this analysis as it was only completed post-therapy and only for those who had received CIRCuiTS^TM^ therapy.

The resulting consensus ranking was subsequently converted into numerical weights using the rank-order centroid (ROC) method. ROC was selected for transparency and feasibility in large samples. It assumes equal spacing between ranks—simplifying trade-offs but ensuring comparability. This approach assigns progressively greater weights to higher-ranked outcomes while ensuring the sum of all weights equals 1.^39^ Three separate sets of ROC weights were constructed based on (1) the aggregate rankings of all participants, (2) rankings provided only by service users, and (3) rankings provided by all staff. All outcome variables were standardized as z-scores. Three weighted composite scores were then calculated at both baseline and post-therapy. Available follow-up data supplemented missing post-therapy outcomes. Missing baseline and post-therapy components of the composite scores were imputed using multivariate imputation by chained equations with predictive mean matching (*k* = 5), stratified by treatment arm to preserve group-specific distributions. Site was included as an auxiliary predictor. Fifty imputed datasets were generated, and estimates were combined using Rubin’s rules. Predictive mean matching was selected to maintain the observed distributional properties of the continuous variables and to minimize the risk of implausible imputed values.^40^

##### Statistical Analysis

To assess treatment effectiveness, we compared groups in the following order: (1) Group cognitive remediation therapy (CRT) vs One-to-One CRT; (2) Independent CRT vs TAU; (3) Group CRT + One-to-One CRT vs TAU. For each comparison we carried out a linear regression model to estimate post-therapy composite scores, adjusting for baseline composite scores and site. Analyses were run using the mi estimate command in STATA to pool results across the imputed datasets.

To assess the robustness of our findings, we conducted a sensitivity analysis using a complete case approach, including only participants with no missing data on all components of the composite scores included in the analysis.

## Results

**Figure 1:**
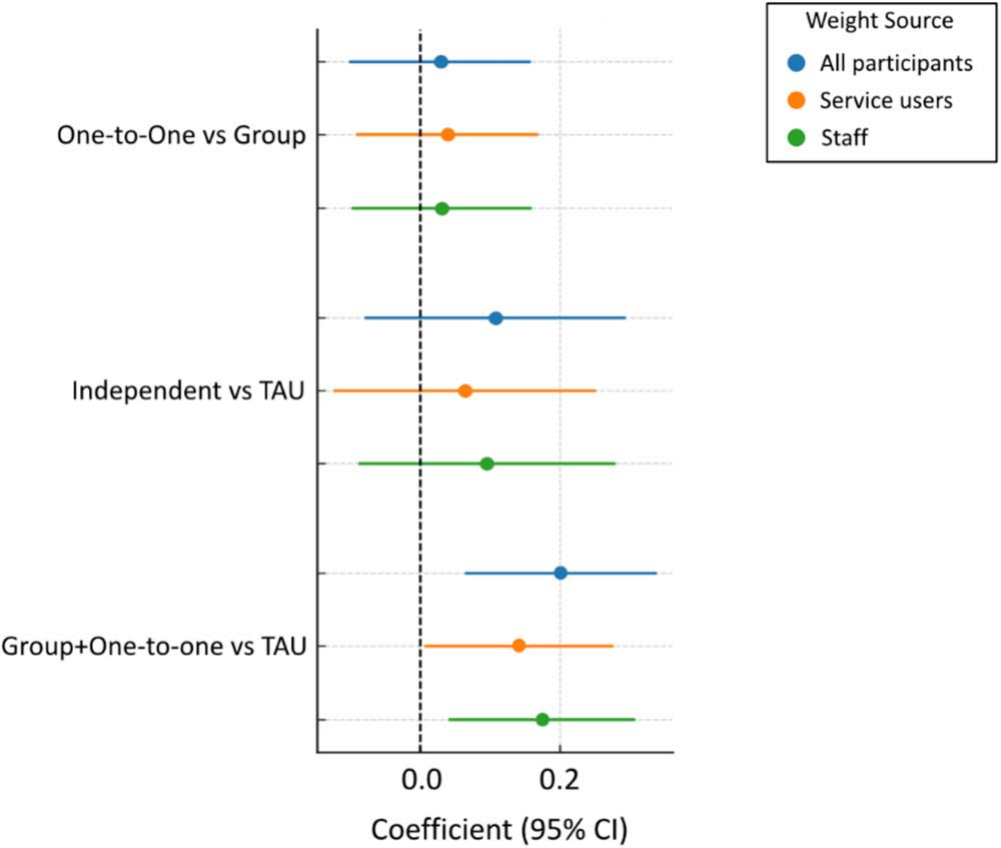
Forest Plot of Post-treatment Improvement Between Intervention Arms Using Outcome Weights Derived from Different Groups.

Figure 1 presents the estimated differences in post-treatment improvement between intervention arms using outcome weights derived from all participants, service users only, and staff only. Across weight sources, the comparison of Group + One-to-One versus TAU showed consistently positive and statistically significant effects (Table 3; all participants: coefficient = 0.201, *P* = .004; service users: coefficient = 0.140, *P* = .041; staff: Coefficient = 0.173, *P* = .011). The other 2 comparisons (One-to-One vs Group and Independent vs TAU) showed smaller coefficients and non-significant differences. We also carried out exploratory analyses of the differences between the TAU arm and separately for the Group and One-to-One arms using multiple imputation. All the comparisons were significant except one, Group vs TAU using the service user composite measure (see Table S14 for the detailed outputs).

**Table 3:** Comparisons of Post-treatment Improvement Between Intervention Arms Using Outcome Weights Derived from Different Groups

Comparison	Coefficient	95% CI	*P*-value
**Using weights derived from all participants**			
One-to-One vs Group	0.029	−0.101 to 0.158	.663
Independent vs TAU	0.107	−0.079 to 0.293	.258
Group+One-to-One vs TAU	0.201	0.064-0.337	**.004** ^**a**^
**Using weights derived from service users only**			
One-to-One vs Group	0.039	−0.091 to 0.169	.556
Independent vs TAU	0.064	−0.123 to 0.251	.498
Group+One-to-One vs TAU	0.140	0.006-0.276	**.041** ^**a**^
**Using weights derived from staff only**			
One-to-One vs Group	0.031	−0.097 to 0.159	.630
Independent vs TAU	0.095	−0.088 to 0.278	.306
Group+One-to-One vs TAU	0.173	0.041-0.306	**.011** ^**a**^

^a^Significant effect.

### Sensitivity Analysis

Complete case analyses yielded results consistent with the primary findings. Across all weighting approaches, Group + One-to-One versus TAU remained the only comparison demonstrating a statistically significant post-treatment improvement, with effect estimates ranging from 0.181 to 0.216 (Tables S15-S17; all participants: 0.216, *P* = .013; service users: 0.181, *P* = .033; staff: 0.187, *P* = .025). All other pairwise comparisons yielded small, non-significant coefficients, with 95% confidence intervals spanning zero.

## Overall Discussion

Our study demonstrates how it is possible to take the views of people with lived experience and their clinicians into account post hoc for completed trials to test treatment benefit using a novel MCDM analysis. This method has rarely been used in health care and never in trials of mental health treatments where the effects are likely to be distributed over primary and secondary outcomes. A recent review suggests that on average there are eight clinical outcomes specified in mental health trials^41^ and the views of service users and clinicians are sometimes included in the choice of these outcomes, but rarely does this influence the choice of the primary outcome.^1-4^ This type of analysis can suggest to clinicians and researchers one of the reasons why a treatment has not been well implemented into services—because it does not fulfil the expectations of users and providers. We thought that there might be differences in ranks of outcome importance based on participants demographic data, and we did find that age and ethnicity were important, and more research is needed to understand how these effects might change the understanding of trial outcomes.

### Does a Weighted Composite Score Produce Different Results to the Original Trial?

The choice of primary outcome for the original CIRCuiTS^TM^ trial, the Global Attainment Scale, was influenced by both clinicians and service users and it is gratifying that the importance of this measure was replicated in the weight given by both stakeholder groups in our survey. It is therefore not surprising that the statistical analysis plan produced the same results as the original trial using the weighted composite score. The result was that CIRCuiTS^TM^ CRT was successful when group+one-to-one therapy was compared to TAU. This same result was also found in the sensitivity analyses using complete case data. This concordance supports the robustness of the observed trial effect and reduces the likelihood that the primary findings are attributable to missing data handling. It also supports the general view that the trial measured benefits appropriately.

There are, however, differences between staff and service users in their attribution of importance for some outcomes despite the high value given to the primary outcome. These were for activities, self-esteem, and quality of life. There was also a difference in the exploratory analyses (Table S14) where group treatment was not statistically significant using the service user weighted composite, although there was a trend. This result tentatively suggests two explanations. First that group treatment may have smaller effects on some components making up the composite and second that for at least some trial participants, the reduction in therapist contact in the group arm has reduced overall benefits. This is not that surprising as there is evidence that for most online psychological therapies the amount of therapist contact is related to outcome benefits and cognitive remediation may be another form of therapy where this effect is also seen. However, this is a subtle difference, but it may have implications for costs. In our clinical opinion a combination of one-to-one therapy at first, followed by group treatment and independent practice might be the most cost-effective clinical service as it combines the clear overall benefits of one-to-one with the cheaper group treatment.

### Use of a Composite Measure in Trials

A recent systematic review suggested that composite measures are underutilized in mental health trials.^41^ Composites are used in cognitive remediation studies, where the primary outcome is often cognition, and measured using a composite of cognitive tests for instance in the MATRICS battery (eg,^42^). However, the individual cognitive domain tests are treated as equivalent for the calculation of the composite rather than weighted by importance. We go further in this paper, with a weighted composite that reflects measures of different target outcome domains important to stakeholders (rather than just a single domain such as cognition). As clinical trials typically rely on a single primary endpoint as the confirmatory measure for an effective intervention, we can potentially make a better and more stakeholder focused decision by incorporating multiple measures that reflect their priorities, weighted by importance.

There are some downsides to this approach. When a continuous composite outcome includes components that do not respond to the intervention, the overall treatment effect can be diluted as these components pull the composite measure toward zero. A weighted composite measure needs an outcome weighting survey that may delay a trial, although researchers could use the same set of weights across different studies as most include the same or similar outcomes. These same weights can also be used post hoc to understand whether the weighted score changes the understanding of different trials and different treatment benefits.

In the future, we advocate that stakeholder views should be incorporated into the trial design and that more emphasis is placed on those views if a single primary outcome is to be used. The new CONSORT reporting guidance^24^ asks a new question on how patients and/or the public were involved in the design, conduct, and/or reporting of the trial. This will encourage the use of stakeholder involvement in trials prospectively so that outcomes will reflect both clinical and service-user priorities and can support the acceptability and implementation of novel therapies into services.

### Strengths and Limitations

This study has multiple strengths such as the inclusion of multiple stakeholder perspectives (including lived experience), an MCDM to integrate values, RCT data and a transparent process for ranking and weighting. The smaller coefficients for the composite mainly reflect the standardization of components because the composite is expressed in SD units rather than GAS points. This does not indicate a smaller underlying effect. The composite showed the same direction and significance as the GAS, suggesting no loss of sensitivity. While composites can sometimes dilute or enhance power depending on the component behavior, this was not the case in our data. We used a simple ranking-based MCDM approach to capture stakeholder priorities, which enabled wide participation, including people with lived experience of psychosis. This method does not allow an estimation of the strength of trade-offs between outcomes. A discrete choice experiment or the use of point allocation might have provided more detailed insight into the relative importance of outcomes but would likely have placed a higher cognitive burden on participants. We also did not ask participants about their background knowledge of cognitive remediation, but it is not a standard NHS treatment, and these staff were not trained to provide it, so we do not think that they will have had much knowledge. It is therefore unlikely to have affected their judgments of outcome importance.

In conclusion, this study highlighted that the primary outcome chosen for the study was valued but there was some disagreement between staff and service users on the value of some secondary outcomes. When importance weights were combined, the trial results were replicated suggesting a robust effect. Taking different perspectives could produce more nuanced results that may help in the generalization and implementation of novel therapies. For this cognitive remediation trial, the only difference was that the benefit of group treatment was not as robust when considering only service user importance weights in an exploratory analysis. However, for all other planned analyses the importance placed by the researchers, the staff, and the service users produced the same effect—a benefit of cognitive remediation. As many trials have not included service user or health care professionals’ views on what outcomes are important, this method may help us understand whether the trial outcomes do show the benefit that stakeholders would like to have seen and is a way of understanding the multidimensional nature of treatment benefit.

## Supplementary Material

sbag006_Revised_Supplementary_data_SchizBul_final_revised

## Data Availability

All data are available either in the supplementary text or from the authors on request.
