# Supplementary material for: Who Should Decide the Outcome for a Clinical Trial? Comparing the Views of Stakeholders on Intervention Benefit Using Multi-Criteria Decision Modelling of Cognitive Remediation
Source: Schizophr Bull. 2026 Apr 21;52(3):sbag006. doi: 10.1093/schbul/sbag006 (PMC13096805; doi:10.1093/schbul/sbag006)
Supplement: sbag006_Revised_Supplementary_data_SchizBul_final_revised [file sbag006_revised_supplementary_data_schizbul_final_revised.docx]

**Supplement: Who chooses the outcome of a clinical trial – people with lived experience, clinical services staff or the researchers? Multi-criterion decision modelling of cognitive remediation outcomes**

Table of Contents

[***Table S1*** Demographic differences across groups 2](#_Toc209447367)

[***Table S2:*** Kruskal-Wallis test results across all three groups 3](#_Toc209447368)

[***Table S3:*** Dunn's post-hoc test across all three groups following the Kruskal-Wallis test 4](#_Toc209447369)

[***Table S4*** Distribution of importance level for each question by group (score of 8 is most important) 5](#_Toc209447370)

[***Table S5:*** Distribution of importance level for each outcome comparing staff groups (8=highly important) 7](#_Toc209447371)

[***Table S6:*** Brown–Forsythe test results for variance across groups 9](#_Toc209447372)

[***Table S7:*** Within-group dispersion of importance level by group 9](#_Toc209447373)

[***Table S8:*** Kruskal-Wallis test results across all groups by ethnicity 10](#_Toc209447374)

[***Table S9:*** Kruskal-Wallis test results across all groups by age 10](#_Toc209447375)

[***Table S10:*** Mann-Whitney U results across all groups by gender 11](#_Toc209447376)

[***Table S11:*** Within group analyses of importance level by age 12](#_Toc209447377)

[***Table S12:*** Within group analyses of importance level by ethnicity 12](#_Toc209447378)

[***Table S13:*** Within group analyses of importance level by gender 13](#_Toc209447379)

[***Table S14.*** Comparisons of post-treatment improvement between intervention arms using outcome weights derived from different groups: primary analysis (multiple imputation) 14](#_Toc209447380)

[***Table S15:*** Comparisons of post-treatment improvement between intervention arms using outcome weights derived from all participants: sensitivity analysis (complete-case) 14](#_Toc209447381)

[***Table S16:*** Comparisons of post-treatment improvement between intervention arms using outcome weights derived from service users only: sensitivity analysis (complete-case) 14](#_Toc209447382)

[***Table S17:*** Comparisons of post-treatment improvement between intervention arms using outcome weights derived from staff only: sensitivity analysis (complete-case) 14](#_Toc209447383)

## ***Table S1*** Demographic differences across groups

We compared age and ethnicity distributions across respondent groups using Mann–Whitney U tests (for age) and chi-square tests (for ethnicity). Service users were significantly younger and less likely to be white compared to staff groups. No demographic differences were observed between providers and managers.

| **Comparison** | **Age (Mann–Whitney U)** | **Ethnicity (χ²)** |
| --- | --- | --- |
| Direct care staff vs. Team Managers | z = -0.76, p = 0.45 | χ² = 0.07, p = 0.79 |
| Direct care staff vs. Service User | **z = 3.19, p < 0.001** | **χ² = 14.09, p < 0.001** |
| Team Managers vs. Service User | **z = 4.19, p < 0.001** | **χ² = 12.35, p < 0.001** |
| All Staff (combined) vs. Service User | **z = 4.26, p < 0.001** | **χ² = 17.22, p < 0.001** |

## ***Table S2:*** Kruskal-Wallis test results across all three groups

| Kruskal-Wallis test ^a^ | | | | | | | | |
| --- | --- | --- | --- | --- | --- | --- | --- | --- |
| Outcome Item | Satisfaction with Therapy | Achieving the service user's individual goals | Increasing the number of things an individual spends time on | Improvement in thinking skills | Increasing self-esteem | Reducing positive symptoms | Improving negative symptoms | Improving overall quality of life |
| Kruskal-Wallis H | 4.21 | 0.64 | 10.63 | 0.64 | 6.78 | 6.58 | 2.74 | 15.67 |
| Asymp. Sig. | 0.12 | 0.72 | **P<0.01^b^** | 0.72 | **0.03^b^** | **0.04^b^** | 0.25 | **P<0.01^b^** |

^a^ Grouping Variable: Three populations within study: Direct care Staff (Group 1), Team Leaders (Group 2), Service users (Group 3)

^b^ P<0.05

## ***Table S3:*** Dunn's post-hoc test across all three groups following the Kruskal-Wallis test for the significant effects found in Table S2

| Dunn's post-hoc test results (with Bonferroni Correction) ^a^ | | | | |
| --- | --- | --- | --- | --- |
| Outcome Item | Group Comparison | Z-value | Raw P-value | Adjusted P-value (Bonferroni) |
| Increasing the number of things an individual spends time on | 1 vs. 2 | -1.78 | 0.04 | 0.11 |
|  | 1 vs. 3 | -3.25 | 0.00 | **P<0.01^b^** |
|  | 2 vs. 3 | -1.48 | 0.07 | 0.21 |
| Increasing self-esteem | 1 vs. 2 | -1.47 | 0.07 | 0.21 |
|  | 1 vs. 3 | 1.13 | 0.13 | 0.38 |
|  | 2 vs. 3 | 2.59 | 0.00 | **0.01**^b^ |
| Reducing positive symptoms | 1 vs. 2 | 2.02 | 0.02 | 0.07 |
|  | 1 vs. 3 | -0.37 | 0.36 | 1 |
|  | 2 vs. 3 | -2.37 | 0.01 | **0.03**^b^ |
| Improving overall quality of life | 1 vs. 2 | 1.70 | 0.04 | 0.13 |
|  | 1 vs. 3 | 3.94 | 0.00 | **P<0.01^b^** |
|  | 2 vs. 3 | 2.26 | 0.01 | **0.04**^b^ |

^a^ Grouping Variable: Three populations within study: Direct care Staff (Group 1), Team Leaders (Group 2), Service users (Group 3)

^b^ P<0.5

## ***Table S4*** Distribution of importance level for each question by group (score of 8 is most important)

Significant differences were observed in the distribution of outcome importance rankings across the three participant groups for improvement in thinking skills, increasing self-esteem, reducing positive symptoms, improving negative symptoms, and satisfaction with therapy (all p < 0.05). Direct care providers differed from service users in their prioritization of improvement in thinking skills, increasing self-esteem, reducing positive symptoms, and satisfaction with therapy. A significant difference between managers and service users was identified only for reducing positive symptoms.

| **Potential outcome** | **Ranking** | **Overall** | **Direct care Staff** | **Team Leaders** | **Service Users** |
| --- | --- | --- | --- | --- | --- |
|  | **No. (%)** | | | | |
| Satisfaction with Therapy | 1 | 33 (35.9%) | 16 (51.6%) | 11 (35.6%) | 6 (20.0%) |
|  | 2 | 13 (14.1%) | 3 (9.7%) | 5 (16.1%) | 5 (16.7%) |
|  | 3 | 8 (8.7%) | 1 (3.2%) | 3 (9.7%) | 4 (13.3%) |
|  | 4 | 6 (6.5%) | 1 (3.2%) | 3 (9.7%) | 2 (6.7%) |
|  | 5 | 4 (4.4%) | 1 (3.2%) | 1 (3.3%) | 2 (6.7%) |
|  | 6 | 11 (12.0%) | 4 (12.9%) | 4 (13.0%) | 3 (10.0%) |
|  | 7 | 5 (5.4%) | 2 (6.5%) | 0 (0.0%) | 3 (10.0%) |
|  | 8 | 12 (13.0%) | 3 (9.7%) | 4 (13.0%) | 5 (16.7%) |
| Achieving the service user's individual goals | 1 | 8 (8.7%) | 1 (3.3%) | 3 (9.7%) | 4 (13.3%) |
|  | 2 | 9 (9.8%) | 2 (6.5%) | 5 (16.1%) | 2 (6.7%) |
|  | 3 | 8 (8.7%) | 6 (19.4%) | 1 (3.2%) | 1 (3.3%) |
|  | 4 | 9 (9.8%) | 2 (6.5%) | 5 (16.1%) | 2 (6.7%) |
|  | 5 | 11 (12.0%) | 5 (16.3%) | 3 (9.7%) | 3 (10.0%) |
|  | 6 | 9 (9.8%) | 2 (6.5%) | 1 (3.2%) | 6 (20.0%) |
|  | 7 | 19 (20.7%) | 5 (16.1%) | 8 (25.8%) | 6 (20.0%) |
|  | 8 | 19 (20.7%) | 8 (25.8%) | 5 (16.1%) | 6 (20.0%) |
| Increasing the number of things an individual spends time on | 1 | 11 (12.0%) | 8 (25.8%) | 1 (3.2%) | 2 (6.7%) |
|  | 2 | 14 (15.2%) | 6 (19.4%) | 6 (19.4%) | 2 (6.7%) |
|  | 3 | 13 (14.1%) | 6 (19.4%) | 4 (12.9%) | 3 (10.0%) |
|  | 4 | 15 (16.3%) | 3 (9.7%) | 7 (22.6%) | 5 (16.7%) |
|  | 5 | 14 (15.2%) | 2 (6.45%) | 7 (22.6%) | 5 (16.7%) |
|  | 6 | 12 (13.0%) | 2 (6.5%) | 3 (9.7%) | 7 (23.3%) |
|  | 7 | 6 (6.5%) | 3 (9.7%) | 2 (6.5%) | 1 (3.3%) |
|  | 8 | 7 (7.6%) | 1 (3.2%) | 1 (3.2%) | 5 (16.7%) |
| Improvement in thinking skills | 1 | 3 (3.3%) | 0 (0.0%) | 2 (6.5%) | 1 (3.3%) |
|  | 2 | 11 (12.0%) | 7 (22.6%) | 3 (9.7%) | 1 (3.3%) |
|  | 3 | 13 (14.1%) | 0 (0.0%) | 7 (22.6%) | 6 (20.0%) |
|  | 4 | 13 (14.1%) | 10 (32.3%) | 0 (0.0%) | 3 (10.0%) |
|  | 5 | 15 (16.3%) | 3 (9.7%) | 6 (19.4%) | 6 (20.0%) |
|  | 6 | 17 (18.5%) | 5 (16.1%) | 5 (16.1%) | 7 (23.3%) |
|  | 7 | 11 (12.0%) | 4 (12.9%) | 4 (12.9%) | 3 (10.0%) |
|  | 8 | 9 (9.8%) | 2 (6.5%) | 4 (12.9%) | 3 (10.0%) |
| Increasing self-esteem | 1 | 6 (6.5%) | 1 (3.2%) | 0 (0.0%) | 5 (16.7%) |
|  | 2 | 13 (14.1%) | 4 (12.9%) | 3 (9.8%) | 6 (20.0%) |
|  | 3 | 13 (14.1%) | 7 (22.6%) | 5 (16.1%) | 1 (3.3%) |
|  | 4 | 18 (19.6%) | 3 (9.7%) | 5 (16.1%) | 10 (33.3%) |
|  | 5 | 20 (21.7%) | 12 (38.7%) | 6 (19.4%) | 2 (6.7%) |
|  | 6 | 7 (7.6%) | 0 (0.0%) | 4 (12.9%) | 3 (10.0%) |
|  | 7 | 9 (9.8%) | 4 (12.9%) | 4 (12.9%) | 1 (3.3%) |
|  | 8 | 6 (6.5%) | 0 (0.0%) | 4 (12.9%) | 2 (6.7%) |
| Reducing positive symptoms | 1 | 8 (8.7%) | 0 (0.0%) | 7 (22.6%) | 1 (3.3%) |
|  | 2 | 9 (9.8%) | 2 (6.5%) | 5 (16.1%) | 2 (6.7%) |
|  | 3 | 13 (14.1%) | 4 (12.9%) | 3 (9.7%) | 6 (20.0%) |
|  | 4 | 12 (13.0%) | 6 (19.4%) | 4 (12.9%) | 2 (6.7%) |
|  | 5 | 8 (8.7%) | 3 (9.7%) | 1 (3.2%) | 4 (13.3%) |
|  | 6 | 14 (15.2%) | 9 (29.0%) | 4 (12.9%) | 1 (3.3%) |
|  | 7 | 15 (16.3%) | 3 (9.7%) | 4 (12.9%) | 8 (26.7%) |
|  | 8 | 13 (14.1%) | 4 (12.9%) | 3 (9.8%) | 6 (20.0%) |
| Improving negative symptoms | 1 | 8 (8.7%) | 1 (3.2%) | 2 (6.5%) | 5 (16.7%) |
|  | 2 | 12 (13.0%) | 5 (16.1%) | 3 (9.78%) | 4 (13.3%) |
|  | 3 | 17 (18.5%) | 5 (16.1%) | 6 (19.4%) | 6 (20.0%) |
|  | 4 | 11 (12.0%) | 3 (9.7%) | 4 (12.9%) | 4 (13.3%) |
|  | 5 | 10 (10.9%) | 2 (6.5%) | 4 (12.9%) | 4 (13.3%) |
|  | 6 | 12 (13.0%) | 6 (19.4%) | 5 (16.1%) | 1 (3.3%) |
|  | 7 | 17 (18.5%) | 8 (25.8%) | 5 (16.1%) | 4 (13.3%) |
|  | 8 | 5 (5.4%) | 1 (3.2%) | 2 (6.5%) | 2 (6.7%) |
| Improving overall quality of life | 1 | 14 (15.2%) | 3 (9.7%) | 5 (16.1%) | 6 (20.0%) |
|  | 2 | 9 (9.8%) | 1 (3.2%) | 1 (3.2%) | 7 (23.3%) |
|  | 3 | 6 (6.5%) | 1 (3.2%) | 2 (6. 5%) | 3 (10.0%) |
|  | 4 | 9 (9.8%) | 3 (9.7%) | 3 (9.7%) | 3 (10.0%) |
|  | 5 | 9 (9.8%) | 2 (6.5%) | 3 (9.7%) | 4 (13.3%) |
|  | 6 | 10 (10.9%) | 2 (6.5%) | 6 (19.5%) | 2 (6.7%) |
|  | 7 | 12 (13.0%) | 4 (12.9%) | 4 (12.9%) | 4 (13.3%) |
|  | 8 | 23 (25.0%) | 15 (48.4%) | 7 (22.6%) | 1 (3.3%) |

## ***Table S5:*** Distribution of importance level for each outcome comparing staff groups (8=highly important)

Significant differences were observed in the distribution of outcome importance rankings across the three participant groups for improvement in thinking skills, increasing self-esteem, reducing positive symptoms, improving negative symptoms, and satisfaction with therapy (all p < 0.05). In pairwise comparisons, combined staff (direct care providers and managers) and service users showed significant differences in rankings for reducing positive symptoms and satisfaction with therapy.

| **Potential outcome** | **Ranking** | **Overall** | **EIS Staff** | **EIS Service User** |
| --- | --- | --- | --- | --- |
|  | **No. (%)** | | | |
| Satisfaction with Therapy | 1 | 33 (35.9%) | 27 (43.6%) | 6 (20.0%) |
|  | 2 | 13 (14.1%) | 8 (12.9%) | 5 (16.7%) |
|  | 3 | 8 (8.7%) | 4 (6.5%) | 4 (13.3%) |
|  | 4 | 6 (6.5%) | 4 (6.5%) | 2 (6.7%) |
|  | 5 | 4 (4.4%) | 2 (3.23%) | 2 (6.7%) |
|  | 6 | 11 (12.0%) | 8 (12.9%) | 3 (10.0%) |
|  | 7 | 5 (5.4%) | 2 (3.2%) | 3 (10.0%) |
|  | 8 | 12 (13.0%) | 7 (11.3%) | 5 (16.7%) |
| Achieving the service user's individual goals | 1 | 8 (8.7%) | 4 (6.5%) | 4 (13.3%) |
|  | 2 | 9 (9.8%) | 7 (11.3%) | 2 (6.67%) |
|  | 3 | 8 (8.7%) | 7 (11.3%) | 1 (3.33%) |
|  | 4 | 9 (9.8%) | 7 (11.3%) | 2 (6.7%) |
|  | 5 | 11 (12.0%) | 8 (12.9%) | 3 (10.0%) |
|  | 6 | 9 (9.8%) | 3 (4.8%) | 6 (20.0%) |
|  | 7 | 19 (20.6%) | 13 (21.0%) | 6 (20.0%) |
|  | 8 | 19 (20.6%) | 13 (21.0%) | 6 (20.0%) |
| Increasing the number of things an individual spends time on | 1 | 11 (12.0%) | 9 (14.5%) | 2 (6.7%) |
|  | 2 | 14 (15.2%) | 12 (19.5%) | 2 (6.7%) |
|  | 3 | 13 (14.1%) | 10 (16.1%) | 3 (10.0%) |
|  | 4 | 15 (16.3%) | 10 (16.1%) | 5 (16.7%) |
|  | 5 | 14 (15.2%) | 9 (14.5%) | 5 (16.7%) |
|  | 6 | 12 (13.0%) | 5 (8.06%) | 7 (23.3%) |
|  | 7 | 6 (6.5%) | 5 (8.1%) | 1 (3.3%) |
|  | 8 | 7 (7.6%) | 2 (3.2%) | 5 (16.7%) |
| Improvement in thinking skills | 1 | 3 (3.3%) | 2 (3.2%) | 1 (3.3%) |
|  | 2 | 11 (12.0%) | 10 (16.1%) | 1 (3.3%) |
|  | 3 | 13 (14.1%) | 7 (11.3%) | 6 (20.0%) |
|  | 4 | 13 (14.1%) | 10 (16.2%) | 3 (10.0%) |
|  | 5 | 15 (16.3%) | 9 (14.6%) | 6 (20.0%) |
|  | 6 | 17 (18.5%) | 10 (16.2%) | 7 (23.3%) |
|  | 7 | 11 (12.0%) | 8 (13.0%) | 3 (10.0%) |
|  | 8 | 9 (9.8%) | 6 (9.7%) | 3 (10.0%) |
| Increasing self-esteem | 1 | 6 (6.5%) | 1 (1.6%) | 5 (16.7%) |
|  | 2 | 13 (14.1%) | 7 (11.3%) | 6 (20.0%) |
|  | 3 | 13 (14.1%) | 12 (19.4%) | 1 (3.3%) |
|  | 4 | 18 (19.6%) | 8 (12.9%) | 10 (33.3%) |
|  | 5 | 20 (21.7%) | 18 (29.0%) | 2 (6.7%) |
|  | 6 | 7 (7.6%) | 4 (6.5%) | 3 (10.0%) |
|  | 7 | 9 (9.8%) | 8 (12.9%) | 1 (3.3%) |
|  | 8 | 6 (6.5%) | 4 (6.5%) | 2 (6.7%) |
| Reducing positive symptoms | 1 | 8 (8.7%) | 7 (11.3%) | 1 (3.3%) |
|  | 2 | 9 (9.8%) | 7 (11.3%) | 2 (6.7%) |
|  | 3 | 13 (14.1%) | 7 (11.3%) | 6 (20.0%) |
|  | 4 | 12 (13.0%) | 10 (16.1%) | 2 (6.7%) |
|  | 5 | 8 (8.7%) | 4 (6.5%) | 4 (13.3%) |
|  | 6 | 14 (15.2%) | 13 (21.0%) | 1 (3.3%) |
|  | 7 | 15 (16.3%) | 7 (11.3%) | 8 (26.7%) |
|  | 8 | 13 (14.1%) | 7 (11.3%) | 6 (20.0%) |
| Improving negative symptoms | 1 | 8 (8.7%) | 3 (4.8%) | 5 (16.7%) |
|  | 2 | 12 (13.0%) | 8 (12.9%) | 4 (13.3%) |
|  | 3 | 17 (18.5%) | 11 (17.7%) | 6 (20.0%) |
|  | 4 | 11 (12.0%) | 7 (11.3%) | 4 (13.3%) |
|  | 5 | 10 (10.9%) | 6 (9.7%) | 4 (13.3%) |
|  | 6 | 12 (13.0%) | 11 (17.7%) | 1 (3.3%) |
|  | 7 | 17 (18.5%) | 13 (21.0%) | 4 (13.3%) |
|  | 8 | 5 (5.4%) | 3 (4.8%) | 2 (6.7%) |
| Improving overall quality of life | 1 | 14 (15.2%) | 8 (12.9%) | 6 (20.0%) |
|  | 2 | 9 (9. 8%) | 2 (3.2%) | 7 (23.3%) |
|  | 3 | 6 (6.5%) | 3 (4.8%) | 3 (10.0%) |
|  | 4 | 9 (9.8%) | 6 (9.7%) | 3 (10.0%) |
|  | 5 | 9 (9.8%) | 5 (8.1%) | 4 (13.3%) |
|  | 6 | 10 (10.9%) | 8 (12.9%) | 2 (6.7%) |
|  | 7 | 12 (13.0%) | 8 (12.9%) | 4 (13.3%) |
|  | 8 | 23 (25.0%) | 22 (35.5%) | 1 (3.3%) |

## ***Table S6:*** Brown–Forsythe test results for variance across groups

The dispersion of importance rankings differed across groups. Service users showed the most consistent importance (Mean IQR = 3.25; Mean MAD = 1.69), while managers exhibited the greatest variability (Mean IQR = 3.88; Mean MAD = 1.75). Providers were intermediate.

| **Group** | **Mean IQR** | **Mean MAD** |
| --- | --- | --- |
| Provider (n = 31) | 3.50 | 1.63 |
| Manager (n = 31) | 3.88 | 1.75 |
| Service user (n = 30) | 3.25 | 1.69 |
| All Staff (combined) | 3.69 | 1.69 |
| Overall (N = 92) | 3.54 | 1.70 |

## ***Table S7:*** Within-group dispersion of importance level by group

Brown–Forsythe tests found no significant variance differences for most questions (all p > 0.05), except for reducing positive symptoms, where variance differed significantly across the three groups (p = 0.041). Combining staff groups and comparing them with service users revealed no evidence of unequal variance (all p > 0.05).

| **Outcome item** | **Staff**  **Median (IQR)** | **Manager Median (IQR)** | **Service user Median (IQR)** | **W0 p-value** |
| --- | --- | --- | --- | --- |
| Satisfaction with Therapy | 1.0 (1.0–6.0) | 2.0 (1.0–5.5) | 3.5 (2.0–6.75) | 0.693 |
| Achieving the service user's individual goals | 5.0 (3.0–7.5) | 5.0 (2.5–7.0) | 6.0 (4.0–7.0) | 0.806 |
| Increasing the number of things an individual spends time on | 3.0 (1.5–4.5) | 4.0 (3.0–5.0) | 5.0 (4.0–6.0) | 0.414 |
| Improvement in thinking skills | 4.0 (4.0–6.0) | 5.0 (3.0–6.5) | 5.0 (3.25–6.0) | 0.351 |
| Increasing self-esteem | 5.0 (3.0–5.0) | 5.0 (3.5–6.5) | 4.0 (2.0–4.75) | 0.570 |
| Reducing positive symptoms | 6.0 (4.0–6.0) | 4.0 (2.0–6.0) | 5.5 (3.0–7.0) | **0.041**^a^ |
| Improving negative symptoms | 5.0 (3.0–7.0) | 5.0 (3.0–6.0) | 3.5 (2.0–5.0) | 0.915 |
| Improving overall quality of life | 7.0 (4.5–8.0) | 6.0 (3.5–7.0) | 3.0 (2.0–5.0) | 0.902 |

^a^ P<0.05

## ***Table S8:*** Kruskal-Wallis test results across all groups by ethnicity

| Kruskal-Wallis test | | | | | | | | |
| --- | --- | --- | --- | --- | --- | --- | --- | --- |
| Outcome Item | Satisfaction with Therapy | Achieving the service user's individual goals | Increasing the number of things an individual spends time on | Improvement in thinking skills | Increasing self-esteem | Reducing positive symptoms | Improving negative symptoms | Improving overall quality of life |
| Asian  Median (IQR) | 2.5 (1.0–5.5) | 4.5 (3.5–6.5) | 4.0 (2.5–5.0) | 5.0 (3.0–6.0) | 6.0 (5.0–7.5) | 3.5 (1.0–5.5) | 3.5 (2.5–6.5) | 6.0 (4.5–7.5) |
| Black  Median (IQR) | 3.0 (2.0–6.0) | 7.0 (4.0–8.0) | 5.0 (3.0–6.0) | 5.0 (4.0–6.0) | 4.0 (2.0–4.0) | 6.0 (3.0–7.0) | 4.0 (2.0–7.0) | 4.0 (2.0–7.0) |
| Mixed  Median (IQR) | 7.5 (3.0–8.0) | 4.5 (2.0–7.0) | 5.0 (2.0–7.0) | 4.0 (3.0–6.0) | 2.5 (1.0–5.0) | 5.0 (4.0–7.0) | 4.0 (3.0–5.0) | 5.0 (4.0–6.0) |
| White  Median (IQR) | 2.0 (1.0–6.0) | 6.0 (3.0–7.0) | 3.0 (2.0–5.0) | 5.0 (3.0–7.0) | 5.0 (3.0–5.0) | 5.0 (3.0–7.0) | 5.0 (3.0–6.0) | 6.0 (3.0–8.0) |
| Other  Median (IQR) | 3.0 (2.0–6.0) | 4.5 (4.0–6.0) | 6.5 (5.0–8.0) | 4.0 (3.0–6.0) | 5.0 (2.0–6.0) | 5.0 (4.0–7.0) | 3.5 (2.0–6.0) | 2.0 (1.0–6.0) |
| Kruskal-Wallis H | 6.59 | 2.12 | 10.6 | 1.37 | 14.02 | 5.30 | 0.83 | 4.66 |
| Asymp. Sig. | 0.15 | 0.71 | **0.03**^a^ | 0.84 | **0.007**^a^ | 0.25 | 0.93 | 0.32 |

^a^ P<0.05

## ***Table S9:*** Kruskal-Wallis test results across all groups by age

| Kruskal-Wallis test | | | | | | | | |
| --- | --- | --- | --- | --- | --- | --- | --- | --- |
| Outcome Item | Satisfaction with Therapy | Achieving the service user's individual goals | Increasing the number of things an individual spends time on | Improvement in thinking skills | Increasing self-esteem | Reducing positive symptoms | Improving negative symptoms | Improving overall quality of life |
| 18–25 years  Median (IQR) | 2.5 (1.0–6.0) | 5.5 (5.0–8.0) | 5.0 (3.0–6.0) | 3.5 (3.0–6.0) | 4.0 (2.0–6.0) | 7.0 (4.0–8.0) | 3.5 (3.0–5.0) | 4.0 (2.0–6.0) |
| 26–35 years  Median (IQR) | 2.0 (1.0–6.0) | 6.0 (3.0–7.0) | 3.0 (2.0–6.0) | 5.0 (3.0–6.0) | 4.0 (3.0–5.0) | 5.0 (3.0–6.0) | 4.0 (3.0–6.0) | 5.5 (3.0–8.0) |
| 36–45 years  Median (IQR) | 3.0 (2.0–6.0) | 5.0 (2.0–7.0) | 5.0 (3.0–6.0) | 5.0 (4.0–7.0) | 4.0 (3.0–6.0) | 5.0 (3.0–7.0) | 4.0 (3.0–7.0) | 5.0 (1.0–8.0) |
| 46–55 years  Median (IQR) | 3.0 (1.0–6.0) | 7.0 (4.0–8.0) | 5.0 (4.0–5.0) | 4.0 (3.0–6.0) | 5.0 (3.0–6.0) | 2.0 (1.0–6.0) | 5.0 (2.0–7.0) | 6.0 (3.0–8.0) |
| 56–65 years  Median (IQR) | 1.0 (1.0–2.0) | 3.0 (3.0–7.0) | 2.0 (1.0–3.0) | 5.0 (4.0–7.0) | 5.0 (5.0–5.0) | 6.0 (4.0–7.0) | 5.5 (4.0–6.0) | 7.0 (5.0–8.0) |
| Kruskal-Wallis H | 3.74 | 3.84 | 10.66 | 4.14 | 2.71 | 8.78 | 1.43 | 5.32 |
| Asymp. Sig. | 0.44 | 0.43 | **0.03^a^** | 0.38 | 0.60 | 0.07 | 0.83 | 0.25 |

^a^ P<0.05

## ***Table S10:*** Mann-Whitney U results across all groups by gender

| Kruskal-Wallis test | | | | | | | | |
| --- | --- | --- | --- | --- | --- | --- | --- | --- |
| Outcome Item | Satisfaction with Therapy | Achieving the service user's individual goals | Increasing the number of things an individual spends time on | Improvement in thinking skills | Increasing self-esteem | Reducing positive symptoms | Improving negative symptoms | Improving overall quality of life |
| Female  Median (IQR) | 2.0 (1.0–5.0) | 5.0 (3.0–7.0) | 4.0 (2.0–6.0) | 5.0 (3.0–6.0) | 4.5 (3.0–6.0) | 5.0 (3.0–7.0) | 5.0 (3.0–7.0) | 6.0 (3.0–8.0) |
| Male  Median (IQR) | 4.0 (1.0–6.0) | 6.0 (3.0–7.0) | 4.0 (3.0–6.0) | 5.0 (3.0–6.0) | 4.0 (2.0–5.0) | 5.5 (3.0–7.0) | 3.0 (2.0–6.0) | 5.0 (2.0–7.0) |
| U-stat | 826.0 | 995.5 | 956.0 | 961.0 | 1149.5 | 1028.0 | 1297.0 | 1200.0 |
| P value | 0.10 | 0.81 | 0.58 | 0.61 | 0.32 | 0.99 | 0.13 | 0.16 |

## ***Table S11:*** Within group analyses of importance level by age

| **Outcome Item** | **Staff younger**  Median (IQR) | **Staff older**  Median (IQR) | **Service users younger**  Median (IQR) | **Service users older**  Median (IQR) | **p-value (staff)** | **p-value (service users)** |
| --- | --- | --- | --- | --- | --- | --- |
| Satisfaction with Therapy | 1.0 (1.0–5.0) | 2.0 (1.0–6.0) | 4.0 (2.0–7.0) | 3.0 (2.0–5.0) | 0.289 | 0.360 |
| Achieving the service user's individual goals | 6.0 (3.0–7.0) | 5.0 (3.0–7.0) | 6.0 (4.0–8.0) | 6.0 (1.0–7.0) | 0.450 | 0.582 |
| Increasing the number of things an individual spends time on | 3.0 (1.0–4.0) | 4.0 (3.0–5.0) | 5.0 (4.0–6.0) | 4.0 (3.0–6.0) | **0.008**^a^ | 0.215 |
| Improvement in thinking skills | 5.0 (3.0–7.0) | 5.0 (3.0–6.0) | 5.0 (3.0–6.0) | 5.0 (4.0–7.0) | 0.777 | 0.448 |
| Increasing self-esteem | 5.0 (3.0–5.0) | 5.0 (3.0–6.0) | 4.0 (2.0–5.0) | 4.0 (2.0–6.0) | 0.539 | 0.659 |
| Reducing positive symptoms | 5.0 (4.0–7.0) | 4.0 (2.0–6.0) | 5.0 (3.0–7.0) | 7.0 (3.0–8.0) | 0.173 | 0.963 |
| Improving negative symptoms | 5.0 (3.0–6.0) | 5.0 (3.0–7.0) | 3.0 (2.0–5.0) | 5.0 (3.0–8.0) | 0.931 | 0.120 |
| Improving overall quality of life | 7.0 (4.0–8.0) | 6.0 (3.0–8.0) | 3.5 (2.0–5.0) | 2.0 (2.0–6.0) | 0.854 | 0.766 |

^a^ P<0.05

## ***Table S12:*** Within group analyses of importance level by ethnicity

| **Outcome item** | **Staff white**  Median (IQR) | **Staff non-white**  Median (IQR) | **Service users white**  Median (IQR) | **Service users non-white**  Median (IQR) | **p-value (staff)** | **p-value (service users)** |
| --- | --- | --- | --- | --- | --- | --- |
| Satisfaction with Therapy | 2.0 (1.0–5.0) | 3.0 (1.0–7.0) | 5.0 (2.0–8.0) | 3.0 (2.0–6.5) | 0.112 | 0.496 |
| Achieving the service user's individual goals | 6.0 (3.0–7.0) | 5.0 (3.0–7.0) | 6.0 (5.0–7.0) | 6.0 (3.0–7.0) | 0.641 | 0.793 |
| Increasing the number of things an individual spends time on | 3.0 (2.0–5.0) | 4.0 (3.0–5.0) | 5.0 (4.0–6.0) | 5.0 (3.5–6.0) | 0.138 | 0.772 |
| Improvement in thinking skills | 5.0 (3.0–7.0) | 5.0 (3.0–6.0) | 4.5 (3.0–6.0) | 5.0 (3.5–6.5) | 0.296 | 0.292 |
| Increasing self-esteem | 5.0 (3.0–5.0) | 5.0 (3.0–6.0) | 3.5 (2.0–4.0) | 4.0 (2.0–5.0) | 0.832 | 0.979 |
| Reducing positive symptoms | 5.0 (3.0–6.0) | 4.0 (2.0–6.0) | 5.0 (3.0–8.0) | 5.5 (3.5–7.0) | 0.269 | 0.916 |
| Improving negative symptoms | 5.0 (3.0–6.0) | 5.0 (3.0–7.0) | 4.0 (2.0–7.0) | 3.5 (2.0–5.0) | 0.904 | 0.814 |
| Improving overall quality of life | 6.0 (4.0–8.0) | 7.0 (5.0–8.0) | 3.0 (1.0–5.0) | 3.0 (2.0–5.5) | 0.506 | 0.636 |

## ***Table S13:*** Within group analyses of importance level by gender

| **Outcome item** | **Staff female**  **Median (IQR)** | **Staff male**  **Median (IQR)** | **Service users female**  **Median (IQR)** | **Service users male**  **Median (IQR)** | **p-value (staff)** | **p-value (service users)** |
| --- | --- | --- | --- | --- | --- | --- |
| Satisfaction with Therapy | 2.0 (1.0–4.5) | 3.0 (1.0–6.0) | 2.0 (1.0–7.0) | 4.5 (2.5–6.5) | 0.655 | 0.296 |
| Achieving the service user's individual goals | 5.0 (3.5–7.0) | 5.5 (3.0–7.0) | 6.0 (3.0–7.0) | 6.0 (4.5–7.0) | 0.931 | 0.824 |
| Increasing the number of things an individual spends time on | 4.0 (2.0–5.0) | 3.0 (2.0–4.0) | 5.0 (3.0–6.0) | 5.0 (4.0–6.5) | 0.536 | 0.755 |
| Improvement in thinking skills | 5.0 (3.0–6.0) | 4.5 (3.0–7.0) | 5.0 (3.0–6.0) | 5.0 (3.5–6.0) | 0.814 | 0.823 |
| Increasing self-esteem | 5.0 (3.0–6.0) | 4.0 (3.0–5.0) | 3.0 (2.0–4.0) | 4.0 (2.0–5.5) | 0.157 | 0.223 |
| Reducing positive symptoms | 4.0 (3.0–6.0) | 5.5 (3.0–7.0) | 6.0 (5.0–8.0) | 5.5 (3.0–7.0) | 0.839 | 0.145 |
| Improving negative symptoms | 5.0 (3.0–7.0) | 4.5 (2.0–6.0) | 5.0 (4.0–7.0) | 3.0 (2.0–4.5) | 0.470 | 0.095 |
| Improving overall quality of life | 6.5 (3.5–8.0) | 6.0 (5.0–8.0) | 4.0 (2.0–5.0) | 2.5 (1.5–5.5) | 0.924 | 0.518 |

***Table S14.*** Comparisons of post-treatment improvement between intervention arms using outcome weights derived from different groups: primary analysis (multiple imputation)

| **Comparison** | **Coefficient** | **95% CI** | **p-value** |
| --- | --- | --- | --- |
| ***Using weights derived from all participants*** | | | |
| One-to-One vs TAU | 0.217 | [0.028, 0.406] | **0.025**^a^ |
| Group vs TAU | 0.215 | [0.029, 0.402] | **0.024**^a^ |
| ***Using weights derived from service users only*** | | | |
| One-to-One vs TAU | 0.189 | [0.002, 0.377] | **0.047**^a^ |
| Group vs TAU | 0.174 | [-0.009, 0.358] | 0.062 |
| ***Using weights derived from staff only*** | | | |
| One-to-One vs TAU | 0.191 | [0.008, 0.374] | **0.040**^a^ |
| Group vs TAU | 0.184 | [0.003, 0.364] | **0.046**^a^ |

^a^ P<0.05

## ***Table S15:*** Comparisons of post-treatment improvement between intervention arms using outcome weights derived from all participants: sensitivity analysis (complete-case)

| **Comparison** | **Coefficient** | **95% CI** | **p-value** |
| --- | --- | --- | --- |
| One-to-One vs Group | 0.001 | [-0.161, 0.163] | 0.988 |
| Independent vs TAU | 0.108 | [-0.119, 0.335] | 0.350 |
| Group+One-to-One vs TAU | 0.216 | [0.047, 0.386] | **0.013**^a^ |
| One-to-One vs TAU | 0.217 | [0.028, 0.406] | **0.025**^a^ |
| Group vs TAU | 0.215 | [0.029, 0.402] | **0.024**^a^ |

^a^ P<0.05

## ***Table S16:*** Comparisons of post-treatment improvement between intervention arms using outcome weights derived from service users only: sensitivity analysis (complete-case)

| **Comparison** | **Coefficient** | **95% CI** | **p-value** |
| --- | --- | --- | --- |
| One-to-One vs Group | 0.015 | [-0.144, 0.174] | 0.856 |
| Independent vs TAU | 0.028 | [-0.196, 0.252] | 0.808 |
| Group+One-to-One vs TAU | 0.181 | [0.014, 0.349] | **0.033**^a^ |
| One-to-One vs TAU | 0.189 | [0.002, 0.377] | **0.047**^a^ |
| Group vs TAU | 0.174 | [-0.009, 0.358] | 0.062 |

^a^ P<0.05

## ***Table S17:*** Comparisons of post-treatment improvement between intervention arms using outcome weights derived from staff only: sensitivity analysis (complete-case)

| **Comparison** | **Coefficient** | **95% CI** | **p-value** |
| --- | --- | --- | --- |
| One-to-One vs Group | 0.008 | [-0.148, 0.164] | 0.921 |
| Independent vs TAU | 0.077 | [-0.142, 0.297] | 0.487 |
| Group+One-to-One vs TAU | 0.187 | [0.023, 0.351] | **0.025**^a^ |
| One-to-One vs TAU | 0.191 | [0.008, 0.374] | **0.040**^a^ |
| Group vs TAU | 0.184 | [0.003, 0.364] | **0.046**^a^ |

^a^ P<0.05
